# Quantitative identification and segmentation repeatability of thoracic spinal muscle morphology

**DOI:** 10.1002/jsp2.1103

**Published:** 2020-07-01

**Authors:** Anoosha Pai S, Honglin Zhang, Jason R. Shewchuk, Bedoor Al Omran, John Street, David Wilson, Majid Doroudi, Stephen H. M. Brown, Thomas R. Oxland

**Affiliations:** ^1^ School of Biomedical Engineering University of British Columbia Vancouver Canada; ^2^ ICORD University of British Columbia Vancouver Canada; ^3^ Centre for Hip Health and Mobility University of British Columbia Vancouver Canada; ^4^ Department of Radiology Vancouver General Hospital Vancouver Canada; ^5^ Department of Orthopaedics University of British Columbia Vancouver Canada; ^6^ Department of Cellular and Physiological Sciences University of British Columbia Vancouver Canada; ^7^ Department of Human Health and Nutritional Sciences University of Guelph Guelph Canada; ^8^ Department of Mechanical Engineering University of British Columbia Vancouver Canada

**Keywords:** erector spinae, magnetic resonance imaging, manual segmentation, muscle morphometry, paraspinal muscle, region of interest, thoracic muscle, transversospinalis, trapezius, upright open MRI

## Abstract

**Objective:**

MRI derived spinal‐muscle morphology measurements have potential diagnostic, prognostic, and therapeutic applications in spinal health. Muscle morphology in the thoracic spine is an important determinant of kyphosis severity in older adults. However, the literature on quantification of spinal muscles to date has been limited to cervical and lumbar regions. Hence, we aim to propose a method to quantitatively identify regions of interest of thoracic spinal muscle in axial MR images and investigate the repeatability of their measurements.

**Methods:**

Middle (T4‐T5) and lower (T8‐T9) thoracic levels of six healthy volunteers (age 26 ± 6 years) were imaged in an upright open scanner (0.5T MROpen, Paramed, Genoa, Italy). A descriptive methodology for defining the regions of interest of trapezius, erector spinae, and transversospinalis in axial MR images was developed. The guidelines for segmentation are laid out based on the points of origin and insertion, probable size, shape, and the position of the muscle groups relative to other recognizable anatomical landmarks as seen from typical axial MR images. 2D parameters such as muscle cross‐sectional area (CSA) and muscle position (radius and angle) with respect to the vertebral body centroid were computed and 3D muscle geometries were generated. Intra and inter‐rater segmentation repeatability was assessed with intraclass correlation coefficient (ICC (3,1)) for 2D parameters and with dice coefficient (DC) for 3D parameters.

**Results:**

Intra and inter‐rater repeatability for 2D and 3D parameters for all muscles was generally good/excellent (average ICC (3,1) = 0.9 with ranges of 0.56‐0.98; average DC = 0.92 with ranges from 0.85‐0.95).

**Conclusion:**

The guidelines proposed are important for reliable MRI‐based measurements and allow meaningful comparisons of muscle morphometry in the thoracic spine across different studies globally. Good segmentation repeatability suggests we can further investigate the effect of posture and spinal curvature on muscle morphology in the thoracic spine.

## Graphical abstract

The quantitative morphology of thoracic spine muscles is important for biomechanical modeling, particularly in spinal deformity. This study presentation novel guidelines for the segmentation of key thoracic spine muscles from MRI.

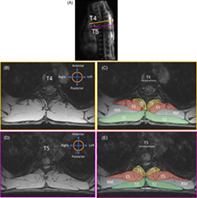

## 1 INTRODUCTION

Spinal muscles are vital to provide mechanical stability and for effective functioning of the spine. Muscular weakness,^1^, ^2^, ^3^ reduction in cross‐sectional area (CSA) and force generating capability,^3^ changes in passive elastic modulus,^4^ and fatty infiltration^5^ are a few muscle‐related factors that may contribute to the onset and development of a number of adult spinal deformities. Thoracic hyperkyphosis affects 20% to 40% of adults over the age of 60,^6^ and is usually accompanied by degenerative loss of lumbar lordosis.^7^ A study on healthy adults (70‐79 years) showed that low density of the paraspinal muscles contributed to kyphosis, beyond the effects of age and osteoporosis.^1^ Furthermore, another study indicated that older men (>65 years) with the smallest paraspinal muscle volume had the largest Cobb angle compared to those with the largest paraspinal muscle size.^8^ Given the rise in aging population (>60 years) from 841 million in 2013 to more than 2 billion in 2050,^9^ the prevalence of adult spinal deformities is increasing. Thus, in vivo assessment of spinal musculature becomes very important in this regard and has implications in diagnostic, prognostic, and therapeutic applications in spinal health.

Computed tomography (CT),^8^, ^10^, ^11^, ^12^, ^13^ and magnetic resonance imaging (MRI)^14^, ^15^, ^16^, ^17^, ^18^, ^19^, ^20^, ^21^ have demonstrated utility in investigating spinal muscles. Although CT typically has higher spatial resolution and shorter scan times, patients have higher radiation exposure and images have lower soft tissue contrast as compared to MRI.^22^ MRI approaches, however, have shown good visualization of skeletal muscle composition^18^, ^23^ along with good reliability for manual segmentation of muscles.^21^ In order to improve clinical relevance and make meaningful comparison of MRI data across different studies, quantification of MRI measurements is crucial.

Most quantitative MRI studies on the paraspinal muscles have only assessed cervical^24^, ^25^, ^26^, ^27^ or lumbar regions.^3^, ^8^, ^17^, ^18^, ^21^ Consequently, the published literature and descriptions available for defining the regions of interest (ROI) of paraspinal muscles have also focused only on the cervical^28^ and lumbar^23^ regions. The paraspinal muscles in the thoracic spine have been understudied to date. Thoracic spinal musculature, however, has considerable clinical relevance. A recent longitudinal study on older men and women (mean age: 61 years) associated larger Cobb angle with smaller CSA and lower muscle to intramuscular fat ratio of the thoracic spine muscles, particularly those situated nearest to the kyphosis curvature.^13^

Difficulties in consistently identifying spinal muscles in the thorax has led to poor repeatability^18^ and slow clinical translation. Currently, to the best of our knowledge, there are no standardized measurement techniques for the thoracic musculature. Thus, in order to fill this gap in literature we aim (a) to develop a systematic methodology to identify and quantify thoracic spinal muscle morphology from continuous axial MR images of thoracic levels and (b) to assess the repeatability of its measurements.

Both, spinal extensor and flexor muscles are important in stabilizing the spine and its posture.^29^ However, spinal extensor muscle strength is shown to be more important for muscular support of the thoracic spine^30^ and is presumed to have the greater clinical significance with respect to thoracic spinal health.^23^, ^31^ Hence, this work focuses on identification of two paraspinal extensor muscles—erector spinae (ES) and transversospinalis (TS) and one posterior muscle—trapezius (TZ).

## 2 MATERIALS AND METHODS

### 2.1 Study participants

Six (five male and one female) healthy participants were recruited (age 26 ± 6 years, height 177 ± 9 cm, and weight 75 ± 10 kg, BMI 24 ± 3 kg/m^2^ with no history of spine conditions).

### 2.2 Image acquisition

**FIGURE 1:**
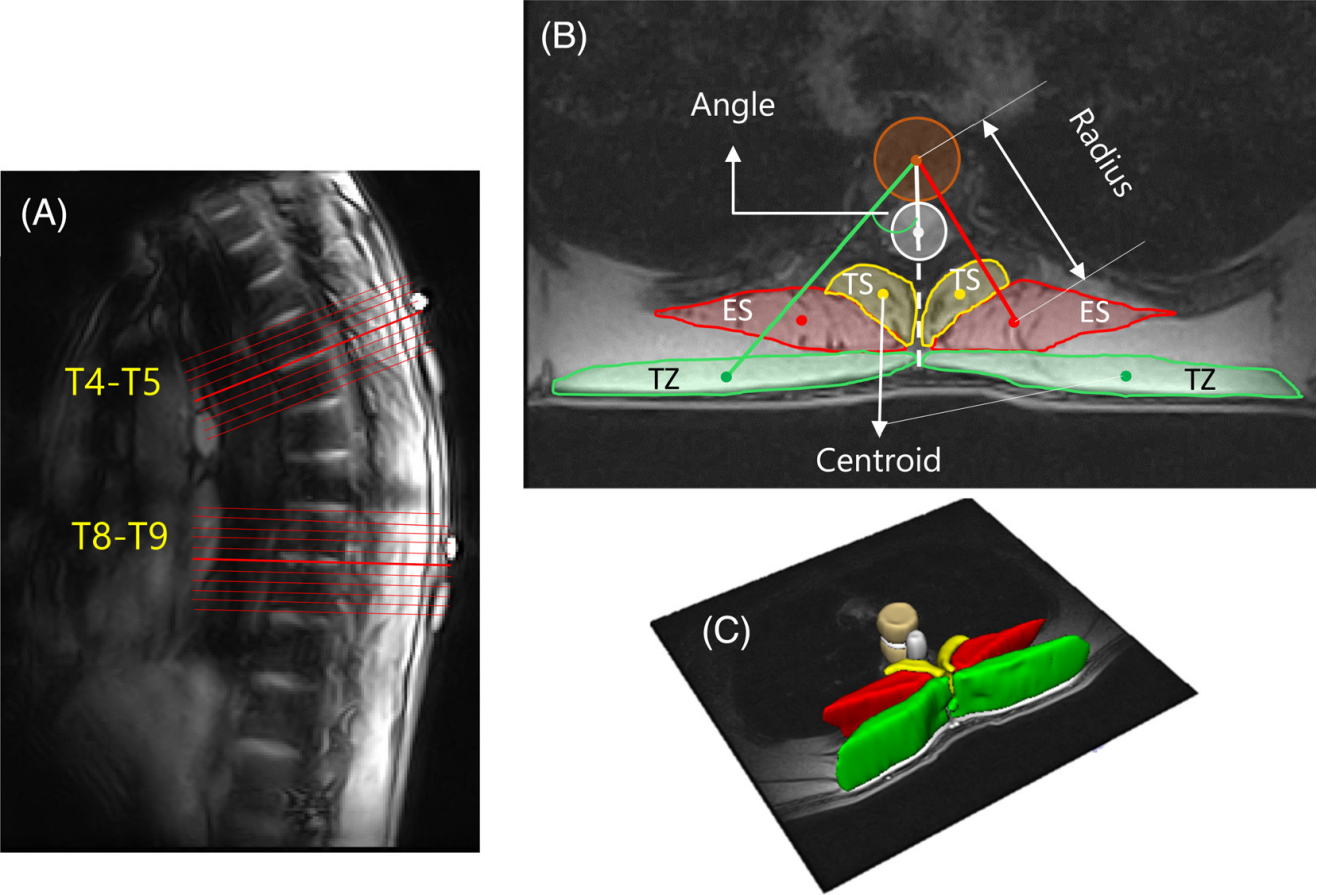
A, Sagittal MR image showing the orientation of the parallel slice stack for thoracic levels T4 to T5 (9 slices) and T8 to T9 (11 slices). B, Image analysis measurements of 2D muscle CSA and position (radius and angle). The brown colored circle represents the vertebral body, the white colored circle represents the vertebral canal. C, 3D geometry obtained from series of 2D muscle segmentations. ES, erector spinae; TS, transversospinalis, TZ, trapezius

The participants were scanned within the 56 cm gap of a 0.5 T vertical open MRI scanner (MROpen, Paramed, Genoa, Italy) using a T1‐weighted Gradient Field Echo sequence (TR/TE = 480/8 ms, FOV 24 cm, scan matrix 224*192, slice thickness 4 mm with 0.4 mm gap, NEX = 2, 153 seconds imaging time). The imaging protocol was approved by the Clinical Research Ethics Board and all the participants signed an informed consent. Two thoracic levels, T4 to T5 (ie, the junction between the upper and the middle thorax), and T8 to T9 (ie, the junction between the middle and lower thorax) were imaged in two separate scans. Images were obtained from a stack of continuous, parallel slices with the middle slice aligned to the center of and parallel to the intervertebral discs (Figure 1A). The number of slices (typically 9 or 11) in a stack was varied in order to cover the entire length of the two vertebral bodies situated on either side of the disc. Towards utilizing the MR scanner to its full capacity and as a groundwork for future studies, the images across both the thoracic levels (T4‐T5 and T8‐T9) were collected for four postures: supine, standing upright, standing with 30° forward flexion in the thorax, and seated with 90°flexion from the hips (sit upright). The data in different postures do not alter or affect the scope of this study and hence statistical comparison of the postural data is beyond the purview of discussion in this article.

#### 2.2.1 Image analysis

3D Slicer (Version 4.10.12, http://www.slicer.org)^32^ was used for image segmentation. Using the descriptive segmentation methodology explained in Section 2.3, 2D CSA of the three muscles was segmented on every slice, and positions (radius and angle) were subsequently computed. Muscle CSA (mm^2^) was determined by manually tracing the outline of the muscle boundary on every slice, and centroid was defined as its geometric center. Angle (degrees) was measured between the line connecting centroids of the muscle and the vertebral body, and the line connecting the centroids of vertebral body and the vertebral canal. Both the vertebral body and the vertebral canal were segmented as circles with constant radii as shown in Figure 1B. Radius (mm) was measured as the distance between the centroids of the muscle and the vertebral body^25^ (Figure 1B). 3D muscle geometry was generated by interpolation of a series of 2D segmented images (Figure 1C).

#### 2.2.2 Data analysis

Segmentation was performed individually by three raters after reviewing guidelines outlined in this article. All the data ((2 levels) × (6 volunteers) × (4 postures) = 48 set of images with each set containing either 9 or 11 slices, avg = 480 slices) were segmented twice by one rater (biomedical engineer, first author), with the second repeated segmentation spaced two  weeks apart. One third of the data (16 set of images, avg = 160 slices) were segmented once by two additional raters (neuro‐radiologist and a neuroradiology fellow, co‐authors). Each rater was initially trained on four data sets (40 slices). The segmentations were reviewed for quality control by the primary rater and those which appeared to be inconsistent and deviated from the guidelines were re‐segmented by corresponding raters. While about 20 out of 40 images were re‐segmented for the training data set, there were no resegmentations for the actual test data set. The segmentations from training data set were excluded from repeatability assessments.

Intra‐rater repeatability was evaluated for two repetitions of the primary rater, while the inter‐rater repeatability was evaluated on one segmentation measure of all three raters. For 2D measures like CSA, radius, and angle, segmentation repeatability for every muscle was assessed using intraclass correlation coefficients ((ICC) (3,1)) computed over all data. ICCs were interpreted as: <0.69 poor, 0.70‐0.79 fair, 0.80‐0.89 good, and 0.90‐1.00 excellent.^33^ For 3D measure, dice coefficient (DC) was used as a statistical validation metric to evaluate the spatial overlap accuracy between two ratings. The value of a DC ranges from 0 to 1, with 0 indicating no spatial overlap between two sets and 1 indicating complete overlap between them.^34^, ^35^ DC >0.70 is considered to be a good overlap in literature.^36^

### 2.3 Defining the ROI

We developed specific guidelines for segmenting ES, TS and TZ from axial MR images using anatomical descriptions of these muscles from the literature. Typical origin and insertions of the individual muscles comprising the three muscle groups are listed in the Table 1. The individual muscles comprising of ES and TS functional groups are epaxial (developed to form the post and paravertebral muscles) and are not encapsulated by an independent layer of epimysium (a factor that helps to discretely delineate a skeletal muscle^23^, ^37^). It is therefore challenging to identify and interpret where the individual fascicles originate and insert, and they are not distinguishable from one another within each muscle group. Thus, the ES and TS are individually identified as single region of interest.

**TABLE 1:** Anatomical attachment sites of trapezius (TZ), erector spinae (ES), and transversospinalis (TS) muscle groups

Muscle group	Muscle/region	Origin	Insertion
TZ ^38^, ^39^	Descending region Transverse region Ascending region	Medial third of superior nuchal line, external occipital protuberance, spinous processes of cervical vertebrae/nuchal ligament. Broad aponeurosis at spinous processes of vertebrae T1 to T4 (or C7‐T3) Spinous process of vertebrae T5 to T12 (or T2‐T12)	Lateral third of clavicle Medial aspect of acromion, superior crest of spine of scapula Medial end of spine of scapula
ES ^44^	***Iliocostalis*** Lumborum Thoracis Cervicis	Common tendon Angle of ribs 6 to 12 Angle of ribs 3 to 7	Angle of ribs 6 to 12 Angle of ribs 1 to 6 Transverse processes of C4 to C6
	***Longissimus*** Lumborum Thoracis Cervicis	Common tendon Transverse processes of T1 to T4 Transverse processes of T1 to T5 and near facet joints C3‐C7	Transverse processes of T1 to T12 Transverse processes of C2 to C6 Mastoid process of temporal bone
	***Spinalis*** Lumborum Thoracis Cervicis	Common tendon Ligamentum nuchae and spinous processes of C7 to T1 Blends with semispinalis capitis	Spinous processes of T1 to T6 Spinous process of C2 Blends with semispinalis capitis
TS ^37^, ^38^, ^41^	***Rotatores*** Longus Brevis Thoracis	Inferior transverse process Upper, posterior part of the inferior transverse process Superior thoracic vertebra	Base of the superior spinous process two levels above Lower border and lateral surface of the superior lamina Inferior thoracic vertebra
	***Multifidus***	Posterior sacrum, posterior superior iliac spine, aponeurosis of the erector spinae, sacroiliac ligament, mammillary processes of the lumbar vertebrae, transverse processes of T1 to T3, articular processes of C4 to C7	Upward and medially to insert along the length of the spinous process spanning two to four segments
	***Semispinalis*** Thoracis Cervicis Capitis	T6 to T10 transverse processes T1 to T5 or T6 transverse processes Superior articular processes C4 or C5 to C7 and the tips of the transverse processes of C7 or T1 to T6 or T7	C6 to T4 spinous processes Spinous process of C2 to C5 Medial part between superior and inferior nuchal lines of the occiput

#### 2.3.1 Trapezius

**FIGURE 2:**
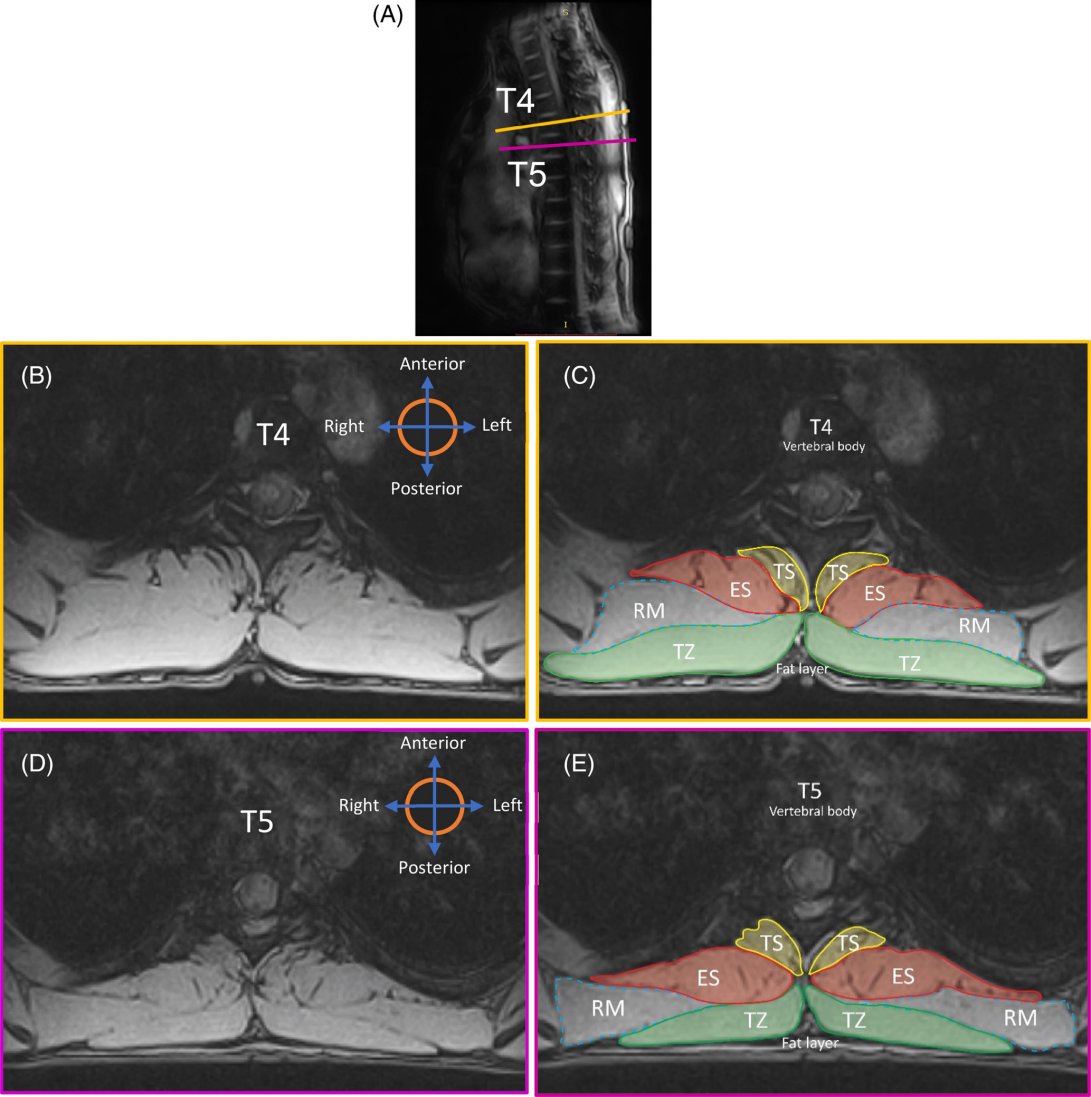
A, Sagittal MRI image showing the slice position and orientation in the thorax. B, Transverse MR image at mid T4 Level. C, Segmentation of the ROI at mid T4. D, Transverse MR image at mid T5 Level. E, Segmentation of the ROI at mid T5 level. ES, erector spinae; RM, rhomboid major; ROI, region of interest; TS, transversospinalis; TZ = trapezius

**FIGURE 3:**
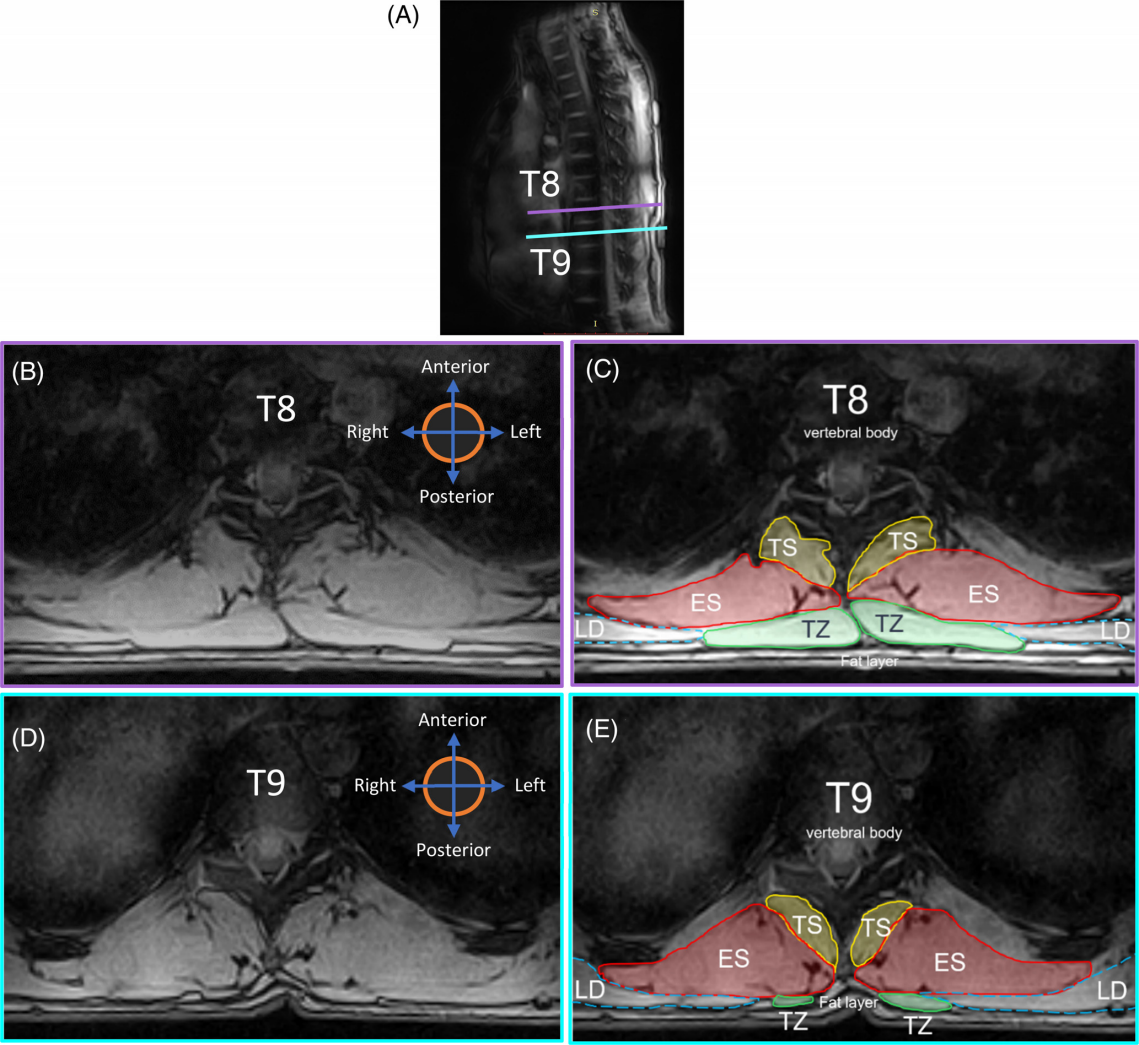
A, Sagittal MRI image showing the slice position and orientation in the thorax. B, Transverse MR image at mid T8 Level. C, Segmentation of the ROI at mid T8. D, Transverse MR image at mid T9 Level. E, Segmentation of the ROI at mid T9 level. ES, erector spinae; LD, latissimus dorsi; ROI, region of interest; TS, transversospinalis; TZ, trapezius

TZ is a flat, triangular muscle that extends over the back of the neck and upper thorax. It is the most superficial muscle that can be located immediately anterior to the subcutaneous fat layer. It can be visualized as a long‐bread or a cigar shaped mass on either side with an increasing volume from the T1 to T3 or T4 levels and decreasing caudally from T4 (Figure 2). The variability in existence and visualization of the TZ in the lower thoracic levels (T8‐T12) is high for different individuals, as the vertebral attachment begins to terminate anywhere between levels T8 and T12.^38^ Furthermore, the shape and size of the TZ in almost all individuals is not expected to be bilaterally symmetric, especially at levels T8 to T9 (Figure 3C). In most transverse slice images, the anterior and posterior borders of the TZ can usually be identified as the longer dimensions of the muscle mass.

1. The anterior border is defined by the fascial line (epimysium) separating TZ and rhomboideus (RH) laterally and TZ and ES group more medially for levels T4 to T5 (Figure 2). For levels T8 to T9, it is the fascial line separating TZ and latissimus dorsi laterally and TZ and ES group more medially (Figure 3).
2. The posterior border for both T4 to T5 and T8 to T9 levels lies abutting the subcutaneous adipose tissue of the back across all levels (Figures 2 and 3).
3. The medial and lateral borders typically reduce to a vertex joining the anterior‐posterior borders. The vertex can either be sharp (Figure 3E) or a rounded contour (medial border in Figure 3C). While the lateral vertex lies surrounded by adjacent subcutaneous adipose tissue, the medial vertex appears to terminate at the supraspinous ligament, seldom distinctly visible in the MR images as a small round projection posterior to the spinous process.

#### 2.3.2 Erector spinae

ES is a large musculotendinous mass that forms the intermediate level of the back muscles. It consists of three long columns of muscles, which from the most medial to the most lateral are named as spinalis, longissimus, and iliocostalis. In an axial MR slice, ES looks like a fish‐shaped muscle mass, with a laterally pointing tail region. The size of ES usually increases along different vertebral levels going craniocaudally.^38^, ^39^

**FIGURE 4:**
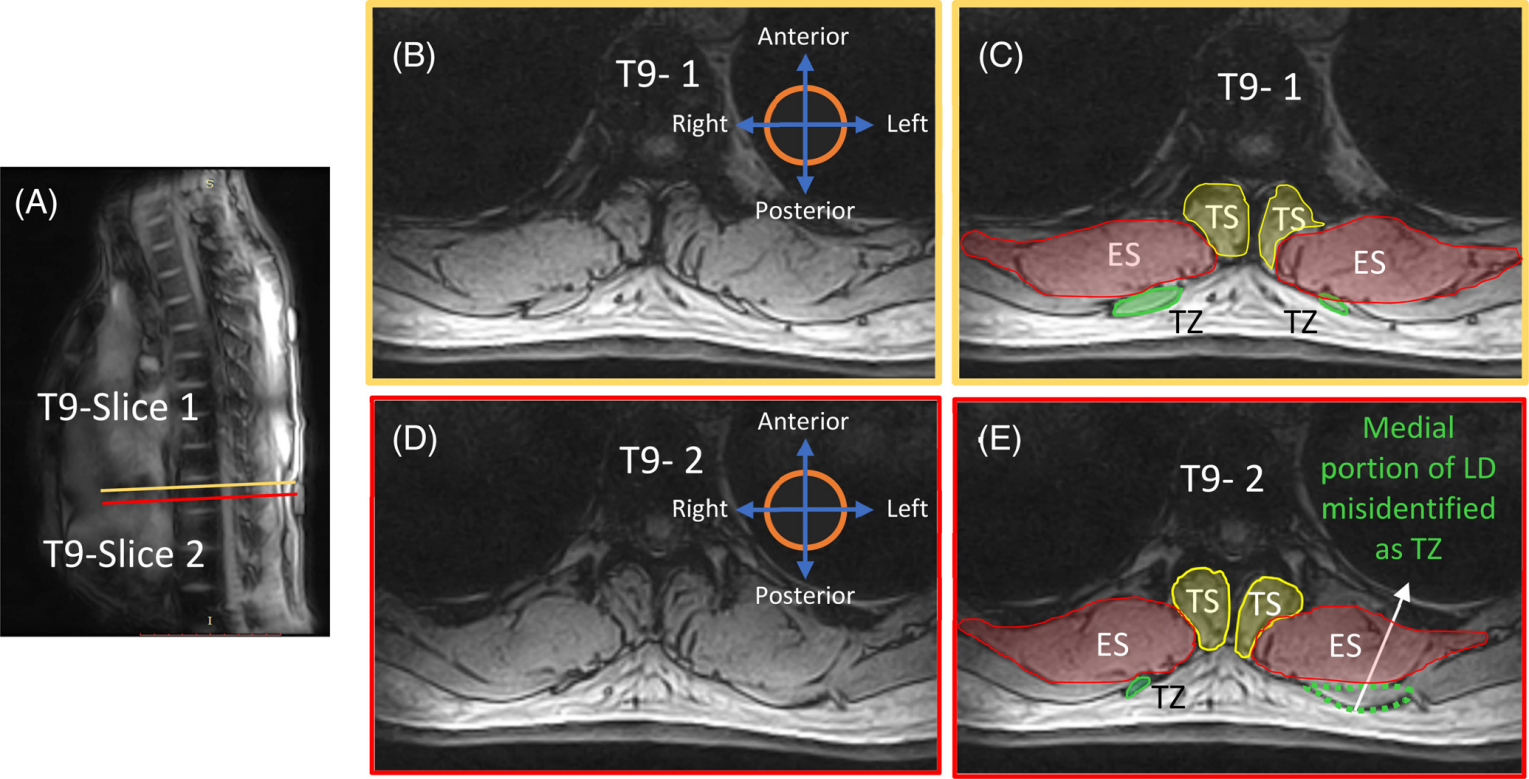
A, Sagittal MRI image showing the slice position and orientation at level T9. B, Transverse MR image of Slice‐1 at level T9. C, Segmentation of the correct ROI for TZ on Slice‐1. D, Transverse MR image of Slice‐2 at level T9. E, Misidentification of LD as ROI of TZ on MR image of Slice‐2 on T9. ES, erector spinae; LD, latissimus dorsi; ROI, region of interest; TS, transversospinalis; TZ, trapezius

1. The anterior border of this muscle group shares most of its medial portion with the lateral fascia of TS throughout the thorax (Figures 2 and 3). The border then deviates laterally tracing the fascial line along the edge of the either a rib or intercostal muscle visible as a contrast in the MR images.
2. The posterior border at higher thoracic levels (T4‐T5) could either be fully shared with the fascial boundary of rhomboideus major (right ES in Figure 2C) or with TZ more medially and with rhomboideus major laterally (left ES in Figure 2C and both ES in Figure 2E). At lower thoracic levels (T8‐T9), however, the anatomy surrounding the posterior border could vary. Normally, the posterior region of the fascicle boundary of ES is shared medially with TZ and laterally with latissimus dorsi (Figure 3C). Nevertheless, in cases where the TZ terminates in any of the lower thoracic vertebrae (T8‐T12), the posterior border of ES could be identified as the fascicle boundary adjacent to subcutaneous adipose tissue medially, TZ in the intermediate portion and latissimus dorsi laterally (Figure 4E).^40^

#### 2.3.3 Transversospinalis

TS is the muscle group lying at the deep layer of intrinsic spinal muscles and consists of semispinalis, multifidus, and rotatores.^41^ TS resembles a bean‐like or a fan‐like shape and is located immediately lateral to the spinous processes of vertebrae (Figures 2 and 3). These muscles span along the entirety of vertebral column typically maintaining a consistent pattern of morphology.^37^, ^40^

1. The medial border across all levels of the thoracic vertebrae predominantly follows the outer edge of the spinous processes deep to where they form the laminae. The border then deviates anterolaterally following the lamina toward the transverse process and ends at some point along the medial edge of the transverse process.^23^
2. The lateral border typically follows the fascial line between ES and TS, identifiable as a contrasted boundary in the MR images. In some cases, mostly at T8 to T9 levels with smaller sized TZ, the lateral border can terminate as a small visible indentation at the subcutaneous adipose tissue posteriorly converging medially to the spinous process (Figure 4).^41^

### 2.4 Technical considerations and segmentation criteria

**FIGURE 5:**
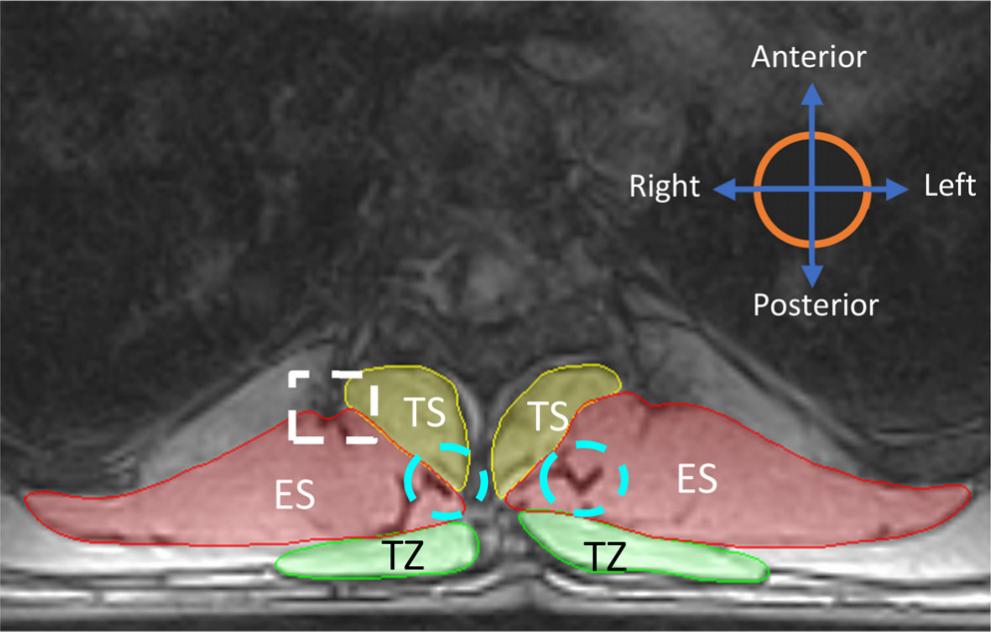
Illustration representing overall inclusion and exclusion criteria while identifying ROI. The white dotted squares represent exclusion criteria. The blue dotted circles represent inclusion criteria. ES, erector spinae; TS, transversospinalis; TZ, trapezius

1. The ROI is segmented as a smooth, continuous island.

- Exclusions: Bony projections (represented by white colored dotted square in Figure 5), fat‐filled tents between the muscle group under consideration and other anatomical structures (eg, fat between ES and RH/LD, TZ, and RH/LD), muscles and surrounding soft tissue (eg, TZ and subcutaneous fat tissue) are excluded from the ROI.
- Inclusions: Muscle extrusions, visible fat‐filled tents or fascia of muscles in the same group (eg, between longissimus and spinalis of the ES group at levels T8 to T9, depicted by cyan color dotted circle on the left in Figure 5) are included. This decision is based on the findings for lumbar spine,^23^, ^42^ where this definition of CSA is shown to have higher clinical relevance as compared to the noninclusive definition. Additionally, fat‐filled tent or gaps between the two muscle groups in consideration (eg, between ES and TS, depicted by cyan colored dotted circle on the right in Figure 5) are included in the ROI of the muscle group having the larger area or size (ES in this, eg). This conclusion was drawn from the fact that addition of the small fat‐filled tent area to the CSA of a relatively bigger muscle would not significantly affect its measurement as much as it would, when added to a muscle whose CSA is smaller or comparable to that of the fat‐tent.

**FIGURE 6:**
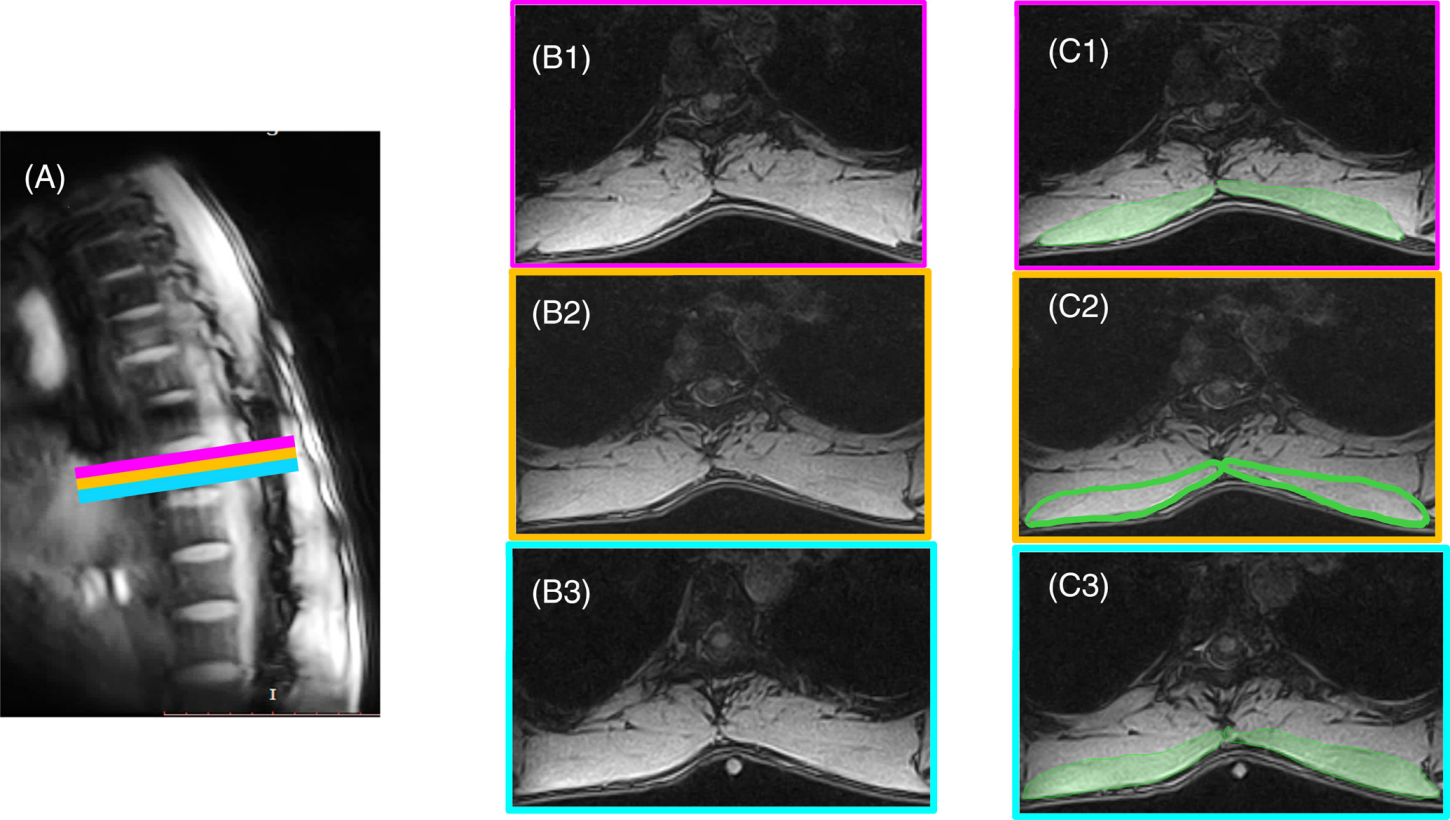
Figure illustrating segmentation method in cases where the muscle has blurred borders. A, Sagittal MRI image showing the three consecutive slices at level T9. B1, Axial image of the superior most slice. B2, Axial image of the middle slice. B3, Axial image of the inferior most slice. C1, Segmentation of TZ on the superior most slice with visible boundary. C2, Segmentation of TZ with unclear border; approximating the anterior border of TZ with segmentation in C1 and C3 as reference. C3, Segmentation of TZ on the inferior most slice with visible boundary. TZ = trapezius

**FIGURE 7:**
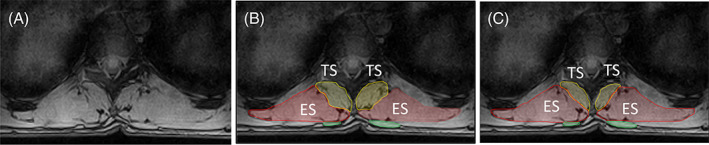
A, Transverse MR image at level T9 showing multiple visible borders between ES and the TS group. B, Approach 1: Segmentation leading to a more concave shape of the ES. C, Approach 2: Segmentation leading to a more convex shape of ES (recommended). ES, erector spinae; TS, transversospinalis

2 Invisible or blurred borders: If the muscle boundary in one or more slices is unclear (usually between TZ and rhomboideus, TZ and latissimus dorsi as shown in Figure 6B, and anterior border of ES with the ribs or intercostal muscles as seen with right ES in Figure 3D), then the muscle boundary is approximated (as in Figure 6C2) to the muscle borders as seen in one or more adjacent slices (Figure 6C1, 6C3) such that, there is no abrupt change in the muscle topology, unless otherwise obvious and distinguishable.
3 Multiple visible borders: If two or more prominent muscle boundaries are visible between two muscle groups, creating confusion and uncertainty about which border to consider while delineating between the two groups (mostly observed for borders between ES and TS as shown in Figure 7A), first choose the border that appears to follow the origin and insertion points of the muscle group across all or most of the slices (for ES in this eg, insertion points must tend toward the spinous process). Next, be sure to identify and mark the same border across all the slices. For slices on which the border is not clear, approximate using segmented images of the preceding slices as reference (as shown in Figure 6). For a stack of continuous slices along the thorax commence the segmentation from the most superior spinal level and move inferior.
4 In cases where there still exists two or more means of identifying the boundaries between two muscle groups (ES and TS), after considering the inclusion/exclusion criteria, and multiple visible border criteria (as shown in Figure 7A), select the border that would make the ROI of the larger muscle group more convex (ES as shown in Figure 7C) than concave (Figure 7B). This assumption is based on observation of general trend in borders of ES and TS in axial MR images.

## 3 RESULTS

### 3.1 Repeatability measures

#### 3.1.1 2‐D measurements

Intra‐rater and inter‐rater segmentation repeatability were generally good/excellent. The average intra‐rater repeatability (ICC (3,1)) for CSA of TZ, ES, and TS was 0.97, 0.93, and 0.83 across levels T4 to T5 and 0.98, 0.97, and 0.92 across levels T8 to T9, respectively. Similarly, the average inter‐rater segmentation repeatability (ICC (3,1)) for CSA of TZ, ES, and TS was 0.97, 0.93, and 0.79 across levels T4‐T5 and 0.98, 0.92, and 0.64 across levels T8‐T9 (Table 2). For radius and angles, it ranged from 0.93 to 1.00, and from 0.83 to 0.99, respectively. The ICCs are summarized in Table 2.

**TABLE 2:** Intra and inter‐rater repeatability of cross‐sectional area (CSA), radius and angle with three raters using intraclass correlation coefficients, ICC (3,1) trapezius (TZ), erector spinae (ES), and transversospinalis (TS)

2D parameters	Muscle	Side	Intra‐rater		Inter‐rater (3 raters)	
			Level		Level	
			T4 to T5	T8 to T9	T4 to T5	T8 to T9
			ICC (3,1)	ICC (3,1)	ICC (3,1)	ICC (3,1)
CSA	TZ	R	0.97	0.98	0.97	0.98
		L	0.97	0.98	0.96	0.98
	ES	R	0.92	0.97	0.93	0.93
		L	0.94	0.97	0.92	0.90
	TS	R	0.81	0.92	0.81	0.71
		L	0.86	0.91	0.76	0.56
Radius	TZ	R	0.99	1.00	0.98	1.00
		L	0.99	1.00	0.98	1.00
	ES	R	0.97	0.99	0.97	0.98
		L	0.99	0.99	0.98	0.97
	TS	R	0.96	0.98	0.93	0.93
		L	0.98	0.98	0.97	0.87
Angle	TZ	R	0.99	0.99	0.99	0.99
		L	0.99	0.99	0.97	0.99
	ES	R	0.93	0.95	0.92	0.92
		L	0.95	0.96	0.93	0.91
	TS	R	0.89	0.92	0.88	0.87
		L	0.90	0.95	0.83	0.89

Abbreviations: L, left; R, right.

#### 3.1.2 3‐D measurements

The average intra‐rater Dice coefficient of volume for TZ, ES, and TS was 0.95, 0.94, and 0.92 across levels T4 to T5 and 0.94, 0.95, and 0.92 across levels T8 to T9, respectively. Similarly, the average inter‐rater Dice coefficient was 0.91, 0.92, and 0.88 across levels T4 to T5 and 0.93, 0.91, and 0.86 across levels T8 to T9 (Table 3).

**TABLE 3:** Intra and inter‐rater repeatability for 3D geometry using dice coefficient (DC) trapezius (TZ), erector spinae (ES), and transversospinalis (TS)

Muscle	Side	Intra‐rater		Inter‐rater	
		Level		Level	
		T4 to T5	T8 to T9	T4 to T5	T8 to T9
TZ	R	0.95	0.94	0.93	0.92
	L	0.94	0.93	0.90	0.90
ES	R	0.94	0.95	0.91	0.93
	L	0.94	0.95	0.90	0.92
TS	R	0.91	0.91	0.86	0.87
	L	0.92	0.92	0.89	0.85

Abbreviations: L, left; R, right.

## 4 DISCUSSION

We developed a systematic methodology to identify and quantify thoracic spinal muscle morphology and assessed the repeatability of its measurements. The segmentation guidelines developed here were based on the available literature,^37^, ^38^, ^39^, ^40^, ^41^ discussions with experts in the field of human anatomy and radiology, and experiential observation. However, there are challenges for consistently and accurately identifying muscle ROI due to a wide variety of variation in human anatomy. Most descriptions for spinal muscle segmentation from axial MR data are based on an image obtained from a single mid‐disc slice. These approaches may lack details regarding the complex three‐dimensional structure of the muscle, changing spatial relationships observed across spinal levels and variability across individuals. The 3D visualization of the anatomy in our study, facilitates more accurate identification of subtle anatomical variations in the muscle morphology, which otherwise may not necessarily be captured with single‐slice images. Some variability observed in muscle identifications along with a few technical considerations for manual segmentation are subsequently outlined.

### 4.1 Variants and remarks

#### 4.1.1 Trapezius

1. The anterior border of TZ can sometimes occur exclusively along the posterior border of rhomboideus and not interact with ES border at all along T4 to T5 (right TZ in Figure 2C).
2. The chances of misidentifying the medial part of latissimus dorsi as TZ, especially caudally along T9 (where TZ has already terminated) is very high as shown in Figure 4B,C. Craniocaudal segmentation is thus recommended to visualize the termination of TZ midway along T8 to T9. When confusion persists in identifying the region of interest, it must be recalled that the volume of TZ always decreases going from level T4/T5 to T12 and hence the region of interest represented by dotted border in Figure 4E cannot be correct.

#### 4.1.2 Erector spinae

**FIGURE 8:**
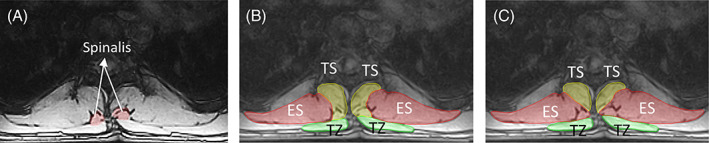
A, Transverse MR image at level T9 showing spinalis muscle of the ES group, appearing like a detached island. B, Incorrect segmentation of ES group, which excludes the spinalis. C, Correct segmentation of ES group, including spinalis. ES, erector spinae; TS, transversospinalis; TZ, trapezius

1. The spinalis, the most medial muscle in the ES group tends to diminish in size as one moves inferiorly, as this muscle extends only in the thoracic region and not in the lumbar (Table 1). The spinalis group can usually appear like small, detached island near the lateral edge of the spinous process, particularly at levels T8 to T9 (Figure 8A). Hence, raters may mistakenly include spinalis in the region of interest of TS, mostly due to its proximity to the TS group, and a faint boundary delineating spinalis with TS. While doing so, raters would likely choose the medial boundary of longissimus muscle as the medial border of the ES group since that border is normally more distinct and prominent in the axial MR images (Figure 8B). In order to avoid this error, the medial border of ES is initially identified (including spinalis if visible) at superior‐most slice and the same border is tracked in subsequent inferior slices (Figure 8C).
2. At levels T8 to T9, for some individuals, it is sometimes observed that the ES appears to be more laterally placed at the spinous process and along the supraspinous ligament (Figure 4) as opposed to the ES seen in Figure 3, which has a more medial position.

#### 4.1.3 Transversospinalis

**FIGURE 9:**
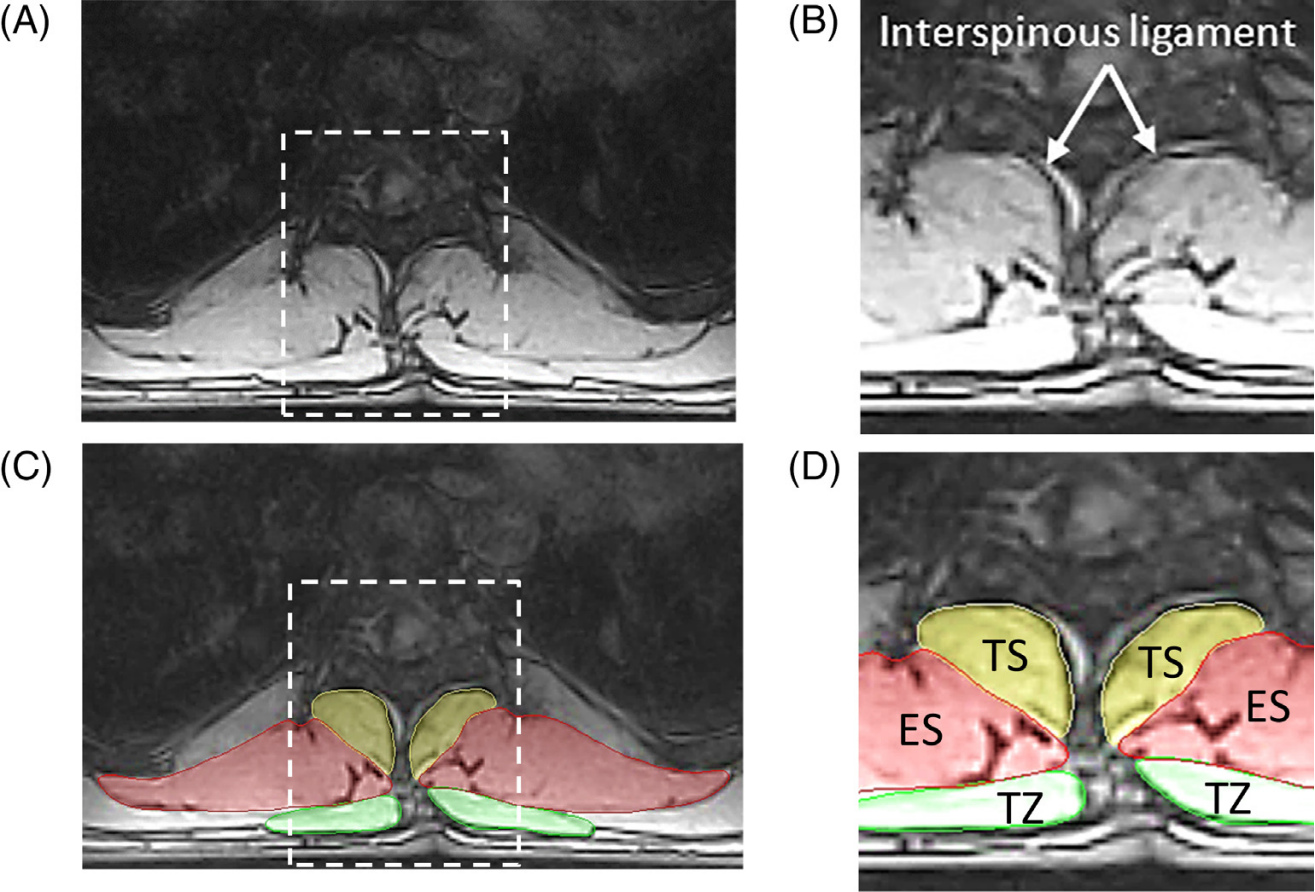
A, Transverse MR image at level T9. B, Zoomed region showing interspinous ligament. C, Transverse MR image at level T9 showing segmented ROIs. D, Zoomed region showing medial border of TS following the interspinous ligament instead of edge of the spinous process. ES, erector spinae; TS, transversospinalis; TZ, trapezius

Sometimes, where the interspinalis muscle and/or the interspinous ligament is evidently distinct with a slightly irregular and brightened edge (Figure 9B), the lateral contour of these must be followed instead of the spinous process in defining the medial border of TS (Figure 9D).^23^

### 4.2 Repeatability measures

Not many studies to the best of our knowledge have assessed repeatability of manual segmentation of the thoracic spinal muscles. Lorbergs et al^13^ looked at segmentation of ES, TS, and TZ at T7 to T8 disc in CT images, and reported both intra and inter rater repeatability to be high (ICC >0.85). Two other MRI studies by Shaikh et al^21^ and Ranson^43^ that looked at combined CSA of ES and multifidus in the lumbar spine reported an average inter‐rater repeatability of 0.82 and 0.96 with ranges of 0.79 to 0.95 and 0.89 to 0.99, respectively.^21^

Overall, our data showed good/excellent intra and inter‐rater repeatability (ICC (3,1)), and the values lie in the typical ballpark found in literature for manual segmentation of spinal muscles. This could reflect that the guidelines provide a step‐by‐step and comprehensive procedure to not only identify the ROI consistently on a series of axial MR images, but also remarks on tackling possible discrepancies the raters might encounter. A lower intra (0.81) and inter‐rater repeatability (0.76) of TS as compared to other muscle groups is attributed to its inherently smaller size, which poses a challenge for precise manual segmentation. The intra and inter‐rater DC values obtained for muscle 3D geometries ranged from 0.85 to 0.95 respectively, which further validates segmentation repeatability. Furthermore, since the CSA is one of the important determinants of spinal muscle health, a standardized quantification method developed in our work could assist to compare and differentiate between healthy and pathological muscles in the thorax. This could also find applications in longitudinal studies, which monitor muscle health of patient with spinal deformities. Moreover, having a uniform segmentation technique could contribute to better inform subject‐specific spine modeling.

### 4.3 Limitations of the study

Interpretation of the data presented in this study has limitations. The MR images of healthy and young individuals were acquired and used in this study while developing the segmentation methodology. The potential use of these guidelines for segmentation of MR images of individuals with a spinal deformity is currently not verified. With the existence of MR scanners of different field strengths, with a variety of different imaging sequences and a large number of variables that influence the image output, the generalizability of the overarching rationales stated in this work for identification of boundaries based on image properties and features is not ascertained. It was also observed that anterolaterally and intricately placed rotatores muscle of TS group could often be included in the region of interest of ES during segmentation due to lower image quality.

## 5 CONCLUSION

The proposed guidelines for identification and quantification of the thoracic musculature extend previously published guidelines from the cervical and lumbar regions. With an increase in uptake of MRI‐based spinal muscle investigations, our study provides a framework for standardization of segmentations of thoracic muscle segmentation. Utilizing a common method of segmentation and more reliable measurements could ultimately lead to clinical translation and contribute in spine modeling.

## CONFLICT OF INTEREST

The authors declare that they have no conflict of interest.

## AUTHOR CONTRIBUTIONS

Anoosha Pai S devised the overall project, the main conceptual ideas, worked out almost all of the technical details, carried out imaging, framed the segmentation guidelines, was the primary rater for segmentation, and performed the numerical calculations. Honglin Zhang assisted in image protocol development and numerical analysis. Jason R. Shewchuk and Bedoor Al Omran were the other trained raters for manual segmentation. John Street provided valuable clinical insights. Majid Doroudi provided insights on the human anatomy portion of the work. Thomas R. Oxland involved in overall planning and supervising the work throughout along with providing crucial feedback and assisting in data analysis. David Wilson and Stephen H. M. Brown provided critical technical feedback, helped shape the research. Anoosha Pai S took the lead in writing the manuscript, with inputs from all authors.

## References

[1] Katzman W , Cawthon P , Hicks GE , Vittinghoff E , Shepherd J . Association of spinal muscle composition and prevalence of hyperkyphosis in healthy community‐dwelling older men and women Wendy. J Gerentol. 2012;67A(2):191‐195. 10.1093/gerona/glr160 PMC3297013 21878482

[2] Mika A , Unnithan VB , Mika P . Differences in thoracic kyphosis and in back muscle strength in women with bone loss due to osteoporosis. Spine (Phila. Pa). 1976;2005(2):241‐246. 10.1097/01.brs.0000150521.10071.df 15644764

[3] Roghani T , Zavieh MK , Manshadi FD , King N , Katzman W . Age‐related hyperkyphosis: update of its potential causes and clinical impacts—narrative review. Aging Clin Exp Res. 2017;29(4):567‐577. 27538834 10.1007/s40520-016-0617-3 PMC5316378

[4] D. P. Zwambag, “Investigation of Intrinsic Spine Muscle Properties to Improve Musculoskeletal Spine Modelling,” Ph.D. dissertation, Dept. of Human Health and Nutritional Sciences, Guelph, Ontario, Canada, 2016.

[5] Crawford RJ , Filli L , Elliott JM , et al. Age‐ and level‐dependence of fatty infiltration in lumbar paravertebral muscles of healthy volunteers. Am J Neuroradiol. 2016;37(4):742‐748. 26635285 10.3174/ajnr.A4596 PMC7960169

[6] Ames CP , Scheer JK , Lafage V , et al. Adult spinal deformity: epidemiology, health impact, evaluation, and management. Spine Deform. 2016;4(4):310‐322. 27927522 10.1016/j.jspd.2015.12.009

[7] Katzman WB , Wanek L , Shepherd JA , Sellmeyer DE . Age‐related Hyperkyphosis: its causes, consequences, and management. J Orthop Sport Phys Ther. 2010;40(6):352‐360. 10.2519/jospt.2010.3099 PMC2907357 20511692

[8] Katzman WB , Miller‐Martinez D , Marshall LM , Lane NE , Kado DM . Kyphosis and paraspinal muscle composition in older men: a cross‐sectional study for the osteoporotic fractures in men (MrOS) research group. BMC Musculoskelet Disord. 2014;15(1):19. 24428860 10.1186/1471-2474-15-19 PMC4029749

[9] United Nations Department of Economic and Social Affairs (2013) World Population Ageing 2013, Retrieved from: http://www.un.org/en/development/desa/population/publications/ageing/WorldPopulationAgeing2013.shtml

[10] Anderson DE , Bean JF , Holt NE , Keel JC . Computed tomography‐based muscle attenuation and electrical impedance myography as indicators of trunk muscle strength independent of muscle size in older adults. Am J Phys Med Rehabil. 2014;93(7):553‐561. 24508931 10.1097/PHM.0000000000000059 PMC4105177

[11] Kumar S . Moment arms of spinal musculature determined from CT scans. Clin Biomech. 1988;3:137‐144. 10.1016/0268-0033(88)90059-9 23915890

[12] Chaffin DB , Redfern MS , Erig M , Goldstein SA . Lumbar muscle size and locations from CT scans of 96 women of age 40 to 63 years. Clin Biomech. Feb. 1990;5(1):9‐16. 10.1016/0268-0033(90)90026-3 23916102

[13] A. L. Lorbergs et al., “A longitudinal study of trunk muscle properties and severity of thoracic kyphosis in women and men: the framingham study,” J Gerontol Ser A Biol Sci Med Sci, 2019. 10.1093/gerona/gly056 PMC6376109 29688268

[14] Jorgensen MJ , Marras WS , Granata KP , Wiand JW . MRI‐derived moment‐arms of the female and male spine loading muscles. Clin Biomech. 2001;16:182‐193. 10.1016/s0268-0033(00)00087-5 11240052

[15] McGill SM , Santaguida L , Stevens J . Measurement of the trunk musculature from T5 to L5 using MRI scans of 15 young males corrected for muscle fibre orientation. Clin Biomech. 1993;8:171‐178. 10.1016/0268-0033(93)90011-6 23915966

[16] Wan Q , Lin C , Li X , Zeng W , Ma C . MRI assessment of paraspinal muscles in patients with acute and chronic unilateral low back pain. 88 1053 London, UK: Br J Radiol; 2015. 10.1259/bjr.20140546 PMC4743557 26105517

[17] Xiao Y , Fortin M , Battié MC , Rivaz H . Population‐averaged MRI atlases for automated image processing and assessments of lumbar paraspinal muscles. Eur Spine J. 2018;27(10):2442‐2448. 30051147 10.1007/s00586-018-5704-z

[18] Mhuiris ÁN , Volken T , Elliott JM , Hoggarth M , Samartzis D , Crawford RJ . Reliability of quantifying the spatial distribution of fatty infiltration in lumbar paravertebral muscles using a new segmentation method for T1‐weighted MRI. BMC Musculoskelet Disord. 2016;17(1):234. 27230072 10.1186/s12891-016-1090-z PMC4882844

[19] Kang CH , Shin MJ , Kim SM , Lee SH , Lee CS . MRI of paraspinal muscles in lumbar degenerative kyphosis patients and control patients with chronic low back pain. Clin Radiol. 2007;62(5):479‐486. 17398274 10.1016/j.crad.2006.12.002

[20] Hwang J , Dufour JS , Knapik GG , et al. Prediction of magnetic resonance imaging‐derived trunk muscle geometry with application to spine biomechanical modeling. Clin Biomech. 2016;37:60‐64. 10.1016/j.clinbiomech.2016.06.001 27337268

[21] Shaikh N et al. The effect of posture on lumbar muscle morphometry from upright MRI. Eur Spine J. 2020. 10.1007/s00586-020-06409-4 32335742

[22] Engelke K , Museyko O , Wang L , Laredo JD . Quantitative analysis of skeletal muscle by computed tomography imaging—state of the art. J Orthopaed Transl. 2018 15:91‐103. 10.1016/j.jot.2018.10.004 PMC6260391 30533385

[23] Crawford RJ , Cornwall J , Abbott R , Elliott JM . Manually defining regions of interest when quantifying paravertebral muscles fatty infiltration from axial magnetic resonance imaging: a proposed method for the lumbar spine with anatomical cross‐reference. BMC Musculoskelet. Disord. 2017;18(25):1‐11. 28103921 10.1186/s12891-016-1378-z PMC5247810

[24] Suderman BL , Vasavada AN . Neck muscle moment arms obtained in‐vivo from MRI: effect of curved and straight modeled paths. Ann Biomed Eng. 2017;45(8):2009‐2024. 28397021 10.1007/s10439-017-1830-8

[25] Stemper BD , Baisden JL , Yoganandan N , Pintar FA , Paskoff GR , Shender BS . Determination of normative neck muscle morphometry using upright MRI with comparison to supine data. Aviat Sp Environ Med. 2010;81(9):878‐882. 10.3357/asem.2758.2010 20824996

[26] Burnett A , O'Sullivan P , Caneiro JP , Krug R , Bochmann F , Helgestad GW . An examination of the flexion‐relaxation phenomenon in the cervical spine in lumbo‐pelvic sitting. J Electromyogr Kinesiol. 2009;19(4):e229‐e236. 18586519 10.1016/j.jelekin.2008.04.015

[27] Anderson JS , Hsu AW , Vasavada AN . Morphology, architecture, and biomechanics of human cervical multifidus. Spine (Phila pa 1976). 2005;30(4):86‐91. 10.1097/01.brs.0000153700.97830.02 15706328

[28] Elliott JM , Cornwall J , Kennedy E , Abbott R , Crawford RJ . Towards defining muscular regions of interest from axial magnetic resonance imaging with anatomical cross‐reference: part II ‐ cervical spine musculature. BMC Musculoskelet Disord. 2018;19(1):171. 29807530 10.1186/s12891-018-2074-y PMC5972401

[29] Kado DM . The rehabilitation of hyperkyphotic posture in the elderly. Eur J Phys Rehabil Med. 2009;45(4):583‐593. 20032918

[30] John W , Ms EJ , Heinz W . Correlation of back extensor strength with thracic kyphosus and lumbar lordosis in estrogen‐deficient women. Am J Phys Med Rehab. 2019;75:370‐374. 10.1097/00002060-199609000-00013 8873705

[31] Lorbergs AL et al. A longitudinal study of trunk muscle properties and severity of thoracic kyphosis in women and men: the Framingham study. J Gerontol Ser A. 2018;74(3):420‐427. 10.1093/gerona/gly056 PMC6376109 29688268

[32] Fedorov A , Beichel R , Kalpathy‐Cramer J , et al. 3D slicer as an image computing platform for the quantitative imaging network. Magn Reson Imaging. 2012;30:1323‐1341. 22770690 10.1016/j.mri.2012.05.001 PMC3466397

[33] Koo TK , Li MY . A guideline of selecting and reporting Intraclass correlation coefficients for reliability research. J Chiropr Med. 2016;15(2):155‐163. 27330520 10.1016/j.jcm.2016.02.012 PMC4913118

[34] Taha AA , Hanbury A . Metrics for evaluating 3D medical image segmentation: analysis, selection, and tool. BMC Med Imaging. 2015 15(1):29. 26263899 10.1186/s12880-015-0068-x PMC4533825

[35] Zou KH . Statistical validation of image segmentation quality based on a spatial overlap index1: scientific reports. Academic Radiology. 2004;11(2):178‐189. 14974593 10.1016/S1076-6332(03)00671-8 PMC1415224

[36] Zijdenbos AP , Dawant BM , Margolin RA , Palmer AC . Morphometric analysis of white matter lesions in MR images: method and validation. IEEE Trans Med Imaging. 1994;13:716‐724. 18218550 10.1109/42.363096

[37] Cornwall J , Stringer MD , Duxson M . Functional morphology of the thoracolumbar transversospinal muscles. Spine (Phila Pa). 2011;36(16):1053‐1061. 10.1097/BRS.0b013e3181f79629 21242870

[38] Netter FH . Atlas of Human Anatomy. 7th ed. Philadelphia, PA: Elsevier; 2018.

[39] Moore KL , Dalley AF , Agur AM . Clinically Oriented Anatomy. 7th ed. Philadelphia, PA: Lippincott Williams & Wilkins; 2014.

[40] Ellis H , Logan BM , Dixon AK , Bowden DJ . Human sectional anatomy: atlas of body sections, CT and MRI images. Boca Raton, FL: CRC Press 2015.

[41] Standring S . Gray's Anatomy. The Anatomical Basis of Clinical Practice. 41st ed. ON, Canada: Elsevier; 2016.

[42] He J , Nakajima T , Espinoza Orias A , An H . Characterizing lumbar multifidus fatty infiltration with MRI: Is there a correct region of interest?. Orthopaedic Research Society Conference; 2016; Orlando, USA, no. Paper 0290.

[43] Ranson CA , Burnett AF , Kerslake R , Batt ME , O'Sullivan PB . An investigation into the use of MR imaging to determine the functional cross sectional area of lumbar paraspinal muscles. Eur Spine J. 2006;15(6):764‐773. 15895259 10.1007/s00586-005-0909-3 PMC3489434

[44] Mansfield PJ , Neumann DA . Structure and function of the vertebral column Essentials of Kinesiology for the Physical Therapist Assistant. 3rd ed. St. Louis, Missouri: Mosby; 2019.

